# Whole Blood Holding Time Prior to Plasma Processing Alters microRNA Expression Profile

**DOI:** 10.3389/fgene.2021.818334

**Published:** 2022-01-14

**Authors:** Sung Hye Kim, David A. MacIntyre, Lynne Sykes, Maria Arianoglou, Phillip R. Bennett, Vasso Terzidou

**Affiliations:** ^1^ Parturition Group, Department of Surgery and Cancer, Institute of Reproductive and Developmental Biology, Imperial College London, London, United Kingdom; ^2^ March of Dimes European Preterm Birth Research Centre, Imperial College London, London, United Kingdom; ^3^ Academic Department of Obstetrics and Gynaecology, Chelsea and Westminster Hospital, London, United Kingdom

**Keywords:** miRNA - microRNA, plasma, biomarker, hemolysis, whole blood

## Abstract

MicroRNAs (miRNAs) can exhibit aberrant expression under different physiological and pathological conditions. Therefore, differentially expressed circulating miRNAs have been a focus of biomarker discovery research. However, the use of circulating miRNAs comes with challenges which may hinder the reliability for their clinical application. These include varied sample collection protocols, storage times/conditions, sample processing and analysis methods. This study focused on examining the effect of whole blood holding time on the stability of plasma miRNA expression profiles. Whole blood samples were collected from healthy pregnant women and were held at 4°C for 30 min, 2 h, 6 h or 24 h prior to processing for plasma isolation. Plasma RNA was extracted and the expression of 179 miRNAs were analyzed. Unsupervised principal component analysis demonstrated that whole blood holding time was a major source of variation in miRNA expression profiles with 53 of 179 miRNAs showing significant changes in expression. Levels of specific miRNAs previously reported to be associated with pregnancy-associated complications such as hsa-miR-150-5p, hsa-miR-191-5p, and hsa-miR-29a-3p, as well as commonly used endogenous miRNA controls, hsa-miR-16-5p, hsa-miR-25-3p, and hsa-miR-223-3p were significantly altered with increase in blood holding time. Current protocols for plasma-based miRNA profiling for diagnostics describe major differences in whole blood holding periods ranging from immediately after collection to 26 h after. Our results demonstrate holding time can have dramatic effects on analytical reliability and reproducibility. This highlights the importance of standardization of blood holding time prior to processing for plasma in order to minimize introduction of non-biological variance in miRNA profiles.

## 1 Introduction

MicroRNAs (miRNAs) are small single-stranded RNA molecules of approximately 19–22 nucleotides in size that regulate gene expression at post-transcriptional level (Cook et al., 2019). As one of the major classes of gene regulatory molecules, they have been shown to play key roles in cell proliferation (Corney et al., 2007), cell differentiation (Wang et al., 2008), apoptosis (Chan et al., 2005), immune response (Wu et al., 2007) and many other physiological processes. Specific miRNA expression profiles are associated with pathological conditions such as cardiovascular diseases, cancers and pregnancy-related complications and may offer diagnostic and therapeutic value (Bracken et al., 2016; Zhou et al., 2018; Cook et al., 2019). MiRNAs are secreted into extracellular spaces where they can be taken up by nearby cells or released into body fluids such as peripheral blood (Valadi et al., 2007; Kosaka et al., 2010). Despite the presence of RNases in blood, miRNAs remain stable and protected from degradation by RNA-binding proteins (Arroyo et al., 2011), specific lipoproteins (Vickers et al., 2011), or via encapsulation within exosomes (Valadi et al., 2007). This high level of stability and the relative ease of accessing miRNAs through blood sampling has led to increased interest in their utility as non-invasive biomarkers for diagnosis, prognosis and prediction of a wide range of pathological conditions. However, there is a lack of consensus in the methodological parameters used when investigating circulating miRNA expression levels. Factors such as types of blood anticoagulant (Kim et al., 2012), level of hemolysis (Kirschner et al., 2011) and plasma storage conditions (Balzano et al., 2015) can alter the expression levels of specific miRNAs. This study aimed to focus on the effects of whole blood holding time, that is storage time until processing for plasma isolation, on miRNA expression profiles.

## 2 Materials and Methods

### 2.1 Blood Collection, Processing and Storage

This study was approved by the London-Stanmore Research Ethics Committee (REC reference number 14/LO/0328) and informed written consent was obtained from all participants. At the time of sampling, peripheral blood samples were collected in four vials of 3 ml BD vacutainers containing EDTA (5.4 mg) from each pregnant woman (*n* = 5) and stored at 4°C immediately after collection. After 30 min, 2 h, 6 h and 24 h of holding at 4°C, plasma was isolated by centrifugation at 3,000 rpm for 10 min at 4°C using Heraeus Megafuge 40R (Thermo scientific). Plasma samples were stored at −80°C until further analysis.

### 2.2 Assessment of Hemolysis

Hemolysis in plasma samples was assessed using three different methods. First, samples were visually inspected with pink discoloration indicative of free-hemoglobin in samples at risk of hemolysis. Secondly, absorbance of free hemoglobin in samples was measured at 414 nm using a NanoDrop™ 1000 spectrophotometer (Thermo scientific) with A_414_ threshold of >0.2 arbitrary units considered hemolysis (Kirschner et al., 2011; Kirschner et al., 2013). Third, the miR ratio of hsa-miR-451a to hsa-miR-23a-3p, also referred to as ΔCq (hsa-miR-23a-3p - hsa-miR-451a) was used as a miRNA-based indicator of hemolysis (Blondal et al., 2013; Shah et al., 2016). For this, RT-qPCR was used to determine the Cq values of hsa-miR-451a and hsa-miR-23a-3p, with a miR ratio >5 considered as moderate and >7 as high risk of hemolysis.

### 2.3 RNA Extraction and RT-qPCR

Plasma samples were centrifuged at 3,000 rpm for 10 min at 4°C using Micro CL17R (Thermo scientific) to remove platelets and microparticle contamination as described in previous literature (Cheng et al., 2013). RNA was extracted from 700 µl plasma using Plasma/Serum Circulating and Exosomal RNA Purification Kit (Slurry-format) (Norgen Biotek). During the lysis step, a spike-in of 5,000 amol of synthetic cel-254 (UGC​AAA​UCU​UUC​GCG​ACU​GUA​GG, Exiqon) was added for normalization of variation in the extraction process. RNA obtained was further purified using Amicon Ultra YM-3 columns (Millipore) to a final volume of 25 µl prior to reverse transcription (RT) as recommended by the manufacturer. Due to the low concentration of cell-free circulating RNAs in biofluids such as plasma, precise estimation of RNA concentration is challenging. In this study, RT was performed using equal volume of RNA originating from the equal volume of plasma as described by previous studies (Blondal et al., 2013; Cook et al., 2019; Butler et al., 2020). RT for plasma miRNAs was performed using 2 µl RNA template in 10 µl total RT reaction volume using miRCURY LNA™ Universal RT miRNA cDNA synthesis kit II (Exiqon), with the addition of 0.625 µl spike-in of synthetic miRNA, UniSp6 (10^8^ copies/µl) (Exiqon) for normalization of variation in the RT process.

Following manufacturer’s instructions, the RT-qPCR master mix was prepared using 80-fold diluted cDNA, ExiLENT SYBR Green master mix (Exiqon) and ROX reference dye. RT-qPCR was performed on ABI StepOnePlus (Life Technologies) at 95°C for 10 min, followed by 45 cycles of 95°C 10 s and 60°C 1 min using the Serum/Plasma Focus miRNA PCR panel (Exiqon) containing LNA™ primers. Raw data were exported from StepOne Software v2.3 (Life Technologies) and imported to LinRegPCR (Heart Failure Research Center) (Ruijter et al., 2009; Ruijter et al., 2013) where Cq values were obtained. Individual Cq values were normalized to inter-plate controls (IPC), extraction spike (cel-254) and RT spike (UniSp6) prior to further analysis to account for any variances during extraction, RT and RT-qPCR processes. The IPCs in each plate is UniSp3, where each well has been designed and manufactured to include pre-aliquoted primers and a pre-defined amount of DNA template, thus variation of IPCs within and between plates are minimal. The IPCs are used to calibrate for differences between PCR plate runs.

### 2.4 Statistical Analyses

The Cq values were imported to SIMCA-P v.15 (Umetrics) for principal component analysis (PCA) and to clustvis (http://biit.cs.ut.ee/clustvis/) for unsupervised hierarchical clustering analysis using the Pearson correlation with Ward linkage. Coefficient of Variance (CV) Analysis was performed in Microsoft Excel. Prior to CV analysis, linearization of Cq values was done using 2^−Cq^ for each sample. For univariate analysis, normality was tested using Kolmogorov.Smirnov test. Multiple comparisons of normally distributed data were analysed using one-way analysis of variance (ANOVA) with *Dunnett’s* or *Tukey’s post hoc* test with GraphPad Prism v.5 (GraphPad Software). Linear regression and two-way ANOVA with *Tukey’s post hoc* test were performed using GraphPad Prism v.9 (GraphPad Software).

## 3 Results

### 3.1 Risk of Hemolysis With Increase in Whole Blood Holding Time Prior to Processing

**FIGURE 1:**
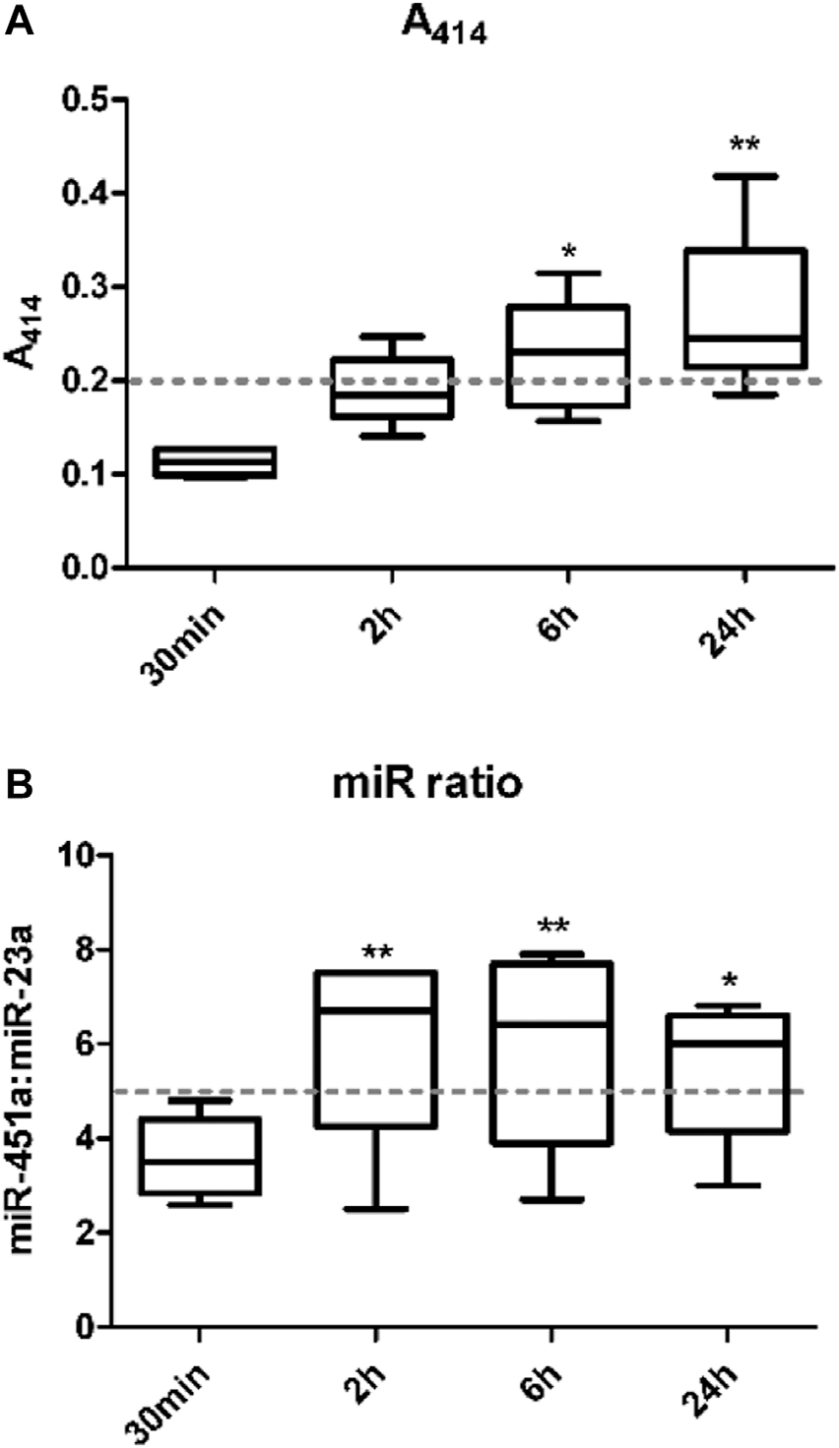
The level of hemolysis is higher with increasing whole blood holding time. Hemolysis in plasma samples was measured. The first method used measurement of the absorbance of hemoglobin at 414 nm using a NanoDrop 1000 spectrophotometer **(A)**. The second method was by examining the ratio of miR-451a to miR-23a-3p (Delta Cq (hsa-miR-23a-3p – hsa-miR-451a)) referred to as miR ratio using RT-qPCR **(B)**.

Hemolysis of red blood cells in whole blood samples has been known to interfere with the quantification of miRNAs (Kirschner et al., 2011; Cheng et al., 2013; Kirschner et al., 2013). Various approaches have been developed to identify the level of hemolysis in a plasma/serum sample. This study investigated the effect of whole blood holding time prior to plasma processing on level/risk of hemolysis using three of these methods including visual inspection, spectrophotometric measurement of hemoglobin absorbance at wavelength 414 nm (A_414_), and the miR ratio of hsa-miR-451a and hsa-miR-23a. There was a significant increase in hemolysis with increase in blood holding time prior to processing. This was detected using both A_414_ (Figure 1A) and miR ratio (Figure 1B) after 6 and 24 h and 2, 6 and 24 h from collection, respectively. The data from individual samples are shown in Supplementary Table S1.

### 3.2 Impact of Blood Holding Time on Plasma miRNA Expression Profile

**FIGURE 2:**
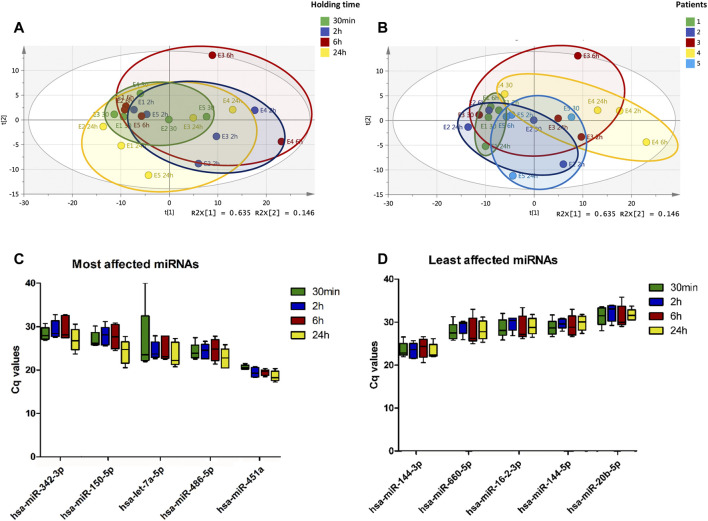
Plasma miRNA expression profile changes with increasing holding time. Whole blood collected from pregnant women were stored at 4°C for 30 min, 2, 6 and 24 h prior to processing for plasma. The expression of plasma miRNAs was determined using RT-qPCR. Principal component analysis (PCA) scores plot of 179 plasma miRNAs colored according to holding time, A 30 min (green), B 2 h (blue), C 6 h (red), and D 24 h (yellow) shows clustering of samples with 30 min holding time **(A)**. PCA shows increase in variability in the plasma miRNA profiles associated with increasing holding time. PCA scores plot of 179 plasma miRNAs colored according to samples, 1 to 5, shows that there is sample-dependent variability in the plasma miRNA profile changes associated with increasing holding time **(B)**. Top 10 miRNAs affected by blood holding time **(C)** and top 10 most stable miRNAs over holding time **(D)** were identified using coefficient of variation.

Principal component analysis (PCA) was performed prior to filtering or statistical analysis to visually inspect clustering patterns of miRNAs profiles following 30 min, 2 h, 6 h and 24 h of holding time at 4°C. Clear differences in the miRNA expression profiles between the time-points were observed with the greatest variation within holding time observed after 2 h (Figure 2A). PCA scores plot also illustrates that both the extent and direction of the changes in these miRNA expression profiles were sample-dependent with some samples being affected more severely than others (Figure 2B).

CV analysis was used to estimate the variation of miRNA expression across different whole blood holding time. In this study, we used the ratio of the standard deviation to the mean of shortest blood holding time point, 30 min, to assess the dispersion of miRNA expression from this time point. Using CVs, the top five miRNAs with most variation over holding time and the top five miRNAs with least variation were identified. MiRNAs whose expression were mostly affected by holding time showed decreases in Cq values with increasing blood holding time (Figure 2C), whereas those less affected remained relatively constant over time (Figure 2D). From the most affected miRNAs, the high variation between samples for hsa-let-7a-5p expression was due to absence of amplification in one of five samples. Notably, hsa-miR-451a showed minimal inter-sample variation which may be due to the fact that this miRNA is significantly affected by the level of hemolysis.

**FIGURE 3:**
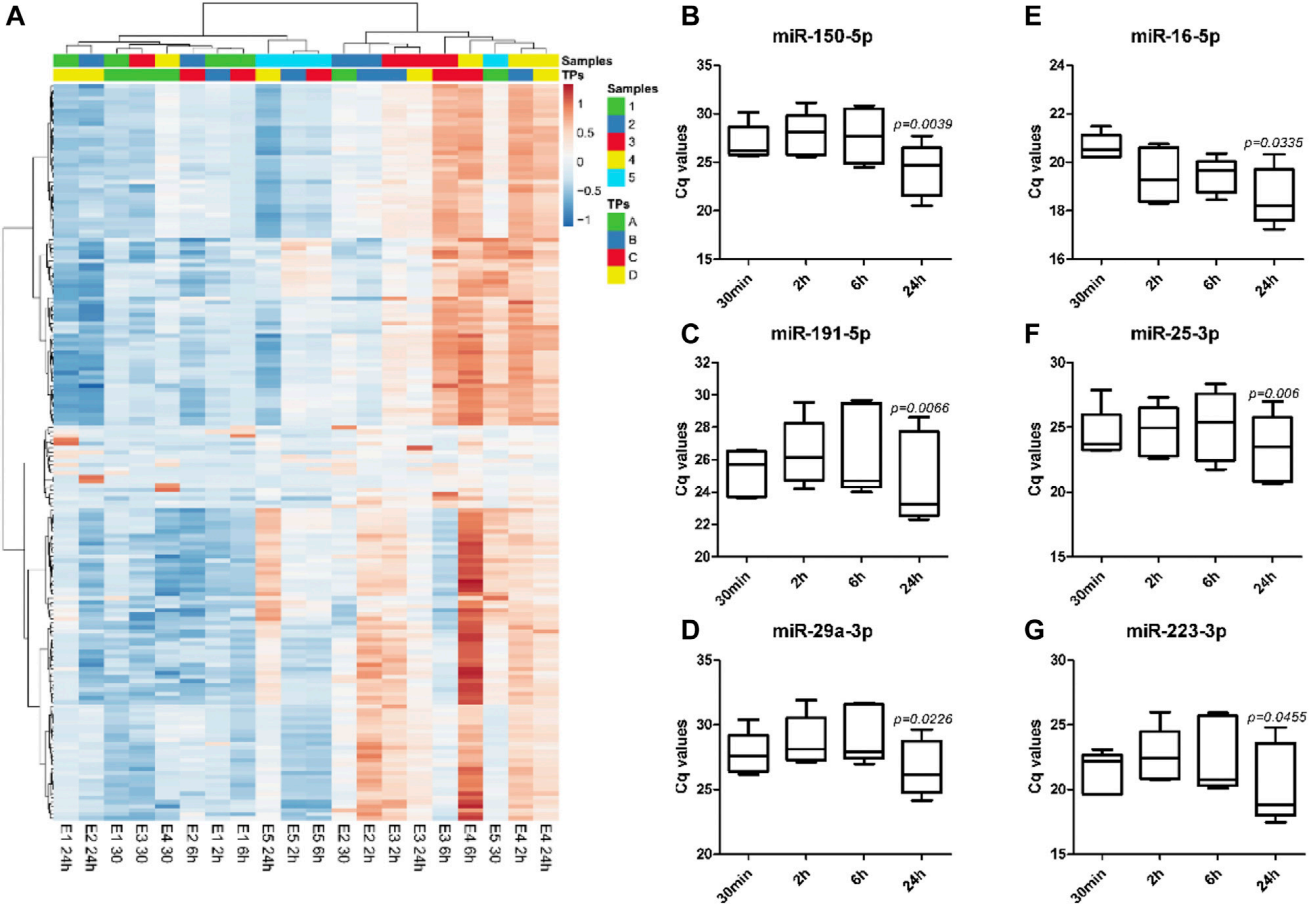
The direction and the extent of changes in miRNA expression profiles with increasing holding time is sample-dependent. Unsupervised hierarchical clustering analysis shows expression profiles of 179 plasma miRNAs in EDTA samples with different holding times (TPs), A: 30 min, B: 2 h, C: 6 h, and D: 24 h **(A)**. Heatmap was built based on Cq values from RT-qPCR using Pearson correlation and Ward linkage. Significant changes in the expression of specific miRNAs associated with preterm birth, miR-150-5p **(B)**, small-for-gestational-age births, miR-191-5p **(C)**, and preeclampsia, miR-29a-3p **(D)**, were observed with increasing holding time. Commonly used endogenous miRNA controls in plasma such as miR-16-5p **(E)**, miR-25-3p **(F)**, and miR-223-3p **(G)**, were also affected by blood holding time.

Analysis of miRNA profile data by unsupervised hierarchical clustering analysis showed major changes in the miRNA expression profiles with different whole blood holding times prior to plasma processing within each sample (Figure 3A). Despite the sample-dependent variance in the effect of blood holding time, two-way ANOVA results showed 53 out of 179 miRNAs were differentially expressed with increasing blood holding time. Among these, hsa-miR-150-5p, hsa-miR-191-5p and hsa-miR-29a-3p which are miRNAs that have been reported to be associated with pregnancy associated pathologies such as preterm birth (Cook et al., 2019), small-for-gestational-age births (Kim et al., 2020) and preeclampsia (Akgör et al., 2021), respectively, were significantly affected by blood holding time. (Figures 3B–D). Similarly, commonly used endogenous plasma miRNA controls, hsa-miR-16-5p, hsa-miR-25-3p and hsa-miR-223-3p (Kroh et al., 2010; Meyer et al., 2010; Guo and Zhang, 2012), were also affected by blood holding time (Figures 3E–G).

### 3.3 Level of Hemolysis and Increase in Whole Blood Holding Time Prior to Processing Affect the Expression of Different Set of miRNAs

**FIGURE 4:**
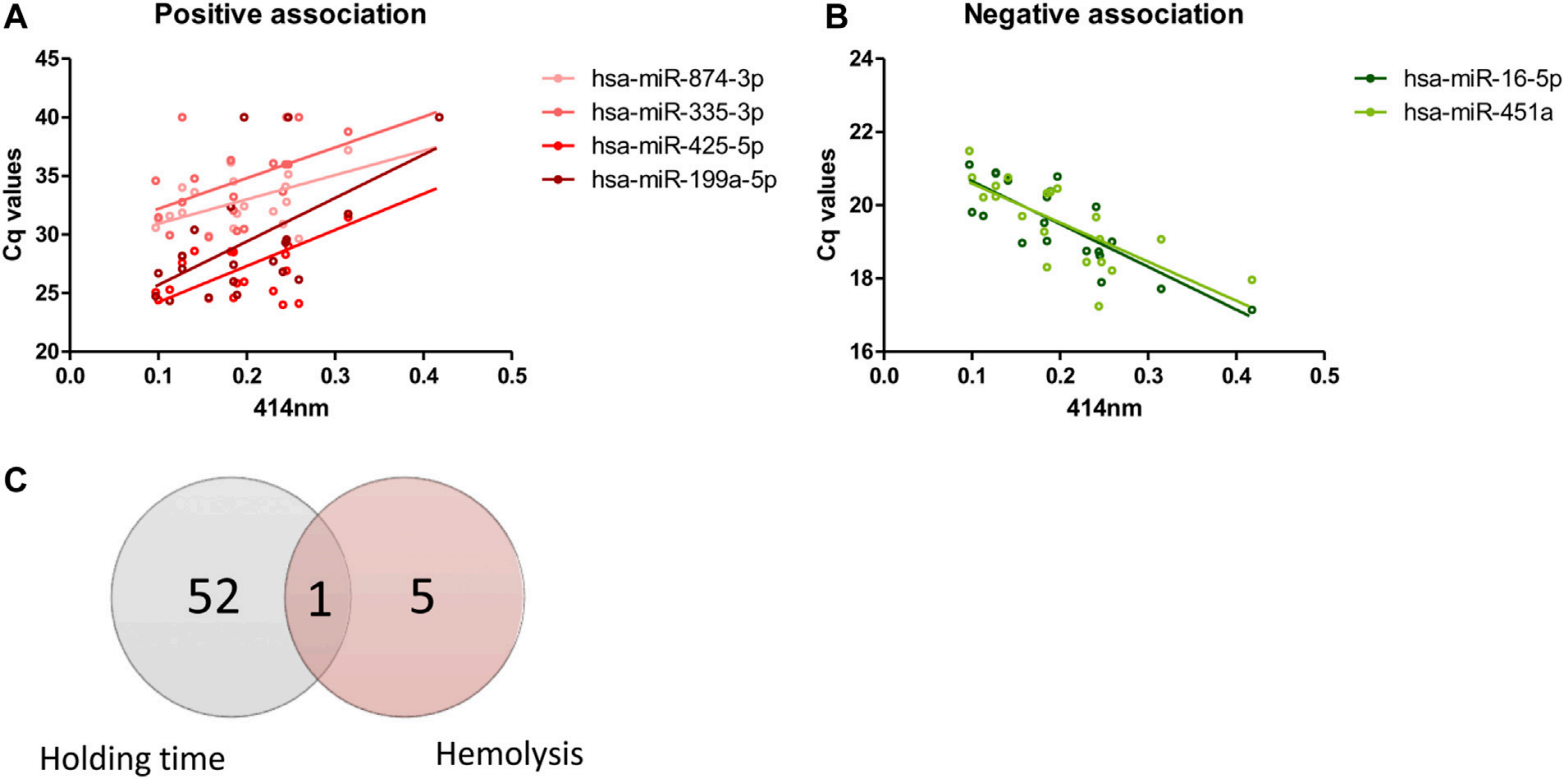
Effects of hemolysis on plasma miRNA expression profiles**.** Risk of hemolysis was found to be higher with increasing blood holding time. Correlation between plasma miRNAs and hemolysis was assessed. The Cq values of four miRNAs demonstrated positive correlation with hemolysis **(A)** and Cq values of two miRNAs demonstrated negative correlation with hemolysis **(B)**. Venn diagram showing the number of miRNAs whose expression is affected by whole blood holding time prior to plasma processing and/or level of hemolysis **(C)**.

From 179 miRNAs examined, six miRNAs demonstrated significant association with level of hemolysis, indicated by absorbance at 414 nm. Of these, four miRNAs showed positive association where increase in Cq values were observed with increasing level of hemolysis (Figure 4A), which indicates decrease in the levels of these miRNAs with increase in hemolysis. Opposite trend was observed in two miRNAs where decrease in Cq values were seen with increase in hemolysis (Figure 4B). Interestingly, only the change in hsa-miR-16-5p expression over blood holding time could be explained by the increase in hemolysis as the majority of miRNAs affected by blood holding time were independent of those affected by hemolysis (Figure 4C).

## 4 Discussion

There is growing attention to circulating miRNAs, specifically plasma-derived, as attractive candidate biomarkers for a variety of pathological conditions. However, they are yet to be used in clinical settings potentially due to the numerous pre-analytical variables affecting most early biomarker studies. Factors such as the type of blood anticoagulant, platelet content, extent of hemolysis, and procedural differences such as blood storage time and conditions until plasma isolation have been reported to affect miRNA expression individually or in combination.

Whole blood samples require processing into plasma or serum prior to miRNA analysis. There are contrasting opinions about the stability of plasma miRNAs under different processing conditions. Some previous studies report high stability of plasma miRNAs for up to 72 h after collection (Mitchell et al., 2008; McDonald et al., 2011), whereas others state that blood holding time up to 4 h significantly affected the levels of specific miRNAs (Wu et al., 2016). These studies focused on a small number of targeted miRNAs from each sample which can mask the effects of prolonged whole blood holding time on the miRNA expression profiles. Our study examined the expression profile of a panel of 179 miRNAs that have been found to be typically present in plasma/serum and were selected based on a combination of experimental data from manufacturers’, in-house studies and results from published, peer-reviewed journals. There were clear changes in plasma miRNA expression profiles with increase in blood holding time prior to processing, significantly affecting the levels of 53 miRNAs. Similar to previous studies, there were no significant changes in the expression of hsa-miR-21-5p, hsa-miR-29b-3p and hsa-miR-1 for up to 24 h, but we did not observe significant increase in hsa-miR-15b-5p and hsa-miR-30e-5p at 2 h as reported by Wu et al. (2016). This may be due to the fact that the extent and the direction of changes in the expression of certain miRNAs in response to holding time appears to be sample-dependent.

The 53 miRNAs significantly affected by blood holding time include miRNAs such as hsa-miR-150-5p, hsa-miR-19b-3p, hsa-miR-23a-3p, hsa-miR-191-5p, 93-5p and hsa-miR-185-5p which have been shown to be predictive of preterm birth (Cook et al., 2019), hsa-miR-191-5p and hsa-miR-107 which are differentially expression in small-for-gestational-age births compared to normal controls (Kim et al., 2020), and hsa-miR-29a-3p, hsa-miR-24-3p, hsa-miR-191-5p, hsa-miR-17-5p and hsa-miR-197-3p which are associated with preeclampsia (Akgör et al., 2021). These changes in miRNA expression with holding time may be due to miRNAs released from red blood cells (RBC). MiRNAs from RBCs and exosomes of RBCs have been found to be dysregulated with holding time (Chen et al., 2018; Huang et al., 2019). Specifically, previous work profiling 52 miRNAs of RBCs have shown that hsa-miR-150-5p and hsa-miR-197-3p levels increase after prolonged blood storage (Kannan and Atreya, 2010). Moreover, there is also increased risk of miRNA degradation with increase in blood holding time prior to plasma processing. These miRNAs have also been identified as potential biomarkers for the prognosis/diagnosis of non-pregnancy-associated conditions. Hsa-miR-150-5p has been found to be associated with multiple pathological conditions such as myasthenia gravis (Sabre et al., 2018), acute myocardial infarction (Li et al., 2019), as well as colorectal cancer (Sarlinova et al., 2016). Similar to this, hsa-miR-29a-3p has been reported to be linked with various types of cancer (Vega et al., 2013; Raeisi et al., 2020) and tuberculosis (Ndzi et al., 2019), and hsa-miR-191-5p (Bahmer et al., 2019) with asthma and chronic kidney disease (Berillo et al., 2020). The top most stable miRNAs included hsa-miR-144, hsa-miR-660-5p, and hsa-miR-20b-5p. The circulating levels of these miRNAs have been associated with various cancers including clear cell renal cell carcinoma, non-small cell lung cancer, and pancreatic cancer (Lou et al., 2017; Qi et al., 2019; Tavano et al., 2020). As the expression of individual miRNAs are differentially affected by the blood holding time prior to plasma processing, it is important to establish the ideal pre-analytical conditions for the specific target miRNA.

Although expression of a wide range of miRNAs were examined in response to pre-processing holding time, this study was limited by its small sample size. Due to the high patient-to-patient variation, it would be important to explore the effects of such pre-analytical variable on miRNA quantification in a larger population. Furthermore, we found a clear increase in hemolysis with increase in whole blood holding time thus it would be important to distinguish miRNAs that are affected by hemolysis and those affected by holding time alone. Another pre-analytical factor that may affect circulating miRNA expression profile is platelet content in plasma samples. There is no international consensus on plasma centrifugation speed, time and temperature when assessing plasma miRNA expression. In this study, we have used single centrifugation step to isolate plasma from whole blood samples, and an additional centrifugation was added prior to analysis to ensure removal of platelet and microparticle contamination. This step has previously shown to minimize the confounding effects of platelets and microparticles prior to archival storage (Cheng et al., 2013), thus allowing use of archived samples from existing cohorts where sample pre-processing was yet to be optimized for miRNA expression profiling.

In conclusion, this study demonstrated clear changes in the plasma miRNA expression profile with varying blood holding time prior plasma processing and highlighted the importance of having a standardized pre-processing protocol for reliable biomarker discovery studies using liquid biopsies.

## Data Availability

The original contributions presented in the study are included in the article/Supplementary Material, further inquiries can be directed to the corresponding author.
