# Supplementary material for: Whole Blood Holding Time Prior to Plasma Processing Alters microRNA Expression Profile
Source: Front Genet. 2022 Jan 14;12:818334. doi: 10.3389/fgene.2021.818334 (PMC8795683; doi:10.3389/fgene.2021.818334)
Supplement: Supplementary file 1 [file Table1.pdf]

Supplementary Table S1. Assessment of haemolysis risk using different methods.

Sample	Visual	A_{414}	miR ratio
E1 30m	No	0.113	3.1
E1 2h	No	0.185	6.7*
E1 6h	No	0.157	5.1*
E1 24h	No	0.418*	5.3*
E2 30m	No	0.127	4.8
E2 2h	No	0.182	7.5*
E2 6h	Yes*	0.241*	7.9*
E2 24h	Yes*	0.259*	6.0*
E3 30m	No	0.097	3.5
E3 2h	No	0.141	6.0*
E3 6h	Yes*	0.230*	7.5*
E3 24h	Yes*	0.245*	6.4*
E4 30m	No	0.100	4.0
E4 2h	Yes*	0.247*	7.5*
E4 6h	Yes*	0.315*	6.4*
E4 24h	Yes*	0.244*	6.8*
E5 30m	No	0.127	2.6
E5 2h	No	0.197	2.5
E5 6h	No	0.189	2.7
E5 24h	No	0.185	3.0

* indicates risk of haemolysis
